# Full-length transcriptome analysis of *Zanthoxylum nitidum* (Roxb.) DC.

**DOI:** 10.7717/peerj.15321

**Published:** 2023-05-04

**Authors:** Yanxia Zhu, Yanfen Huang, Kunhua Wei, Junnan Yu, Jianping Jiang

**Affiliations:** 1 Guangxi Key Laboratory of Medicinal Resources Protection and Genetic Improvement, Guangxi Botanical Garden of Medicinal Plants, Nanning, China; 2 ChongQing Jinzhi Quality Certification Co., LTD, Chongqing, China; 3 Guangxi Key Laboratory for High-quality Formation and Utilization of Dao-di Herbs, Guangxi Botanical Garden of Medicinal Plants, Nanning, China

**Keywords:** *Zanthoxylum nitidum*, PacBio sequencing, Full-length transcriptome, Genome assembly, SSR

## Abstract

*Zanthoxylum nitidum* (Roxb.) DC. (*Z. nitidum*) is a type of Chinese Dao-di herb, also called Liangmianzhen, which is widely used to treat arthralgia, rheumatic arthralgia, and stomach pain. However, genomic resources for *Z. nitidum* are still scarce. This study provides transcriptomic resources for *Z. nitidum* by applying single-molecule real-time (SMRT) sequencing technology. In total, 456,109 circular consensus sequencing (CCS) reads were generated with a mean length of 2,216 bp from *Z. nitidum* roots, old stems, young branches, leaves, flowers, and fruits. Of these total reads, 353,932 were full-length nonchimeric (FLNC) reads with an average length of 1,996 bp. A total of 16,163 transcripts with a mean length of 1,171 bp were acquired. Of these transcripts, 14,231 (88%) were successfully annotated using public databases. Across all the 16,163 transcripts, we identified 6,255 long non-coding RNAs (lncRNAs) and 22,780 simple sequence repeats (SSRs). Furthermore, 3,482 transcription factors were identified. Among the SSR loci, 1–3 nucleotide repeats were dominant, occupying 99.36% of the total SSR loci, with mono-, di-, and tri-nucleotide repeats accounting for 61.80%, 19.89%, and 5.02% of the total SSR loci, respectively. A total of 36 out of 100 randomly selected primer pairs were verified to be positive, 20 of which showed polymorphism. These findings enrich the genetic resources available for facilitating future studies and research on relevant topics such as population genetics in *Z. nitidum*.

## Introduction

*Zanthoxylum nitidum* (Roxb.) DC. (*Z. nitidum*) is a famous Chinese Dao-di herb, also named Liangmianzhen, which is mainly distributed in southern China, specifically in Guangxi, Guangdong, and Hainan. Its root has been found to be effective in treating stomach aches, toothaches, rheumatic arthralgia, traumatic injury, and venomous snake bites (State Pharmacopoeia Commission of the PRC, 2020). Liangmianzhen is also an ingredient in many patented Chinese medicines, such as “Dieda Wanhua oil,” “SanjiuWeitai granules,” and “Huoluo Zhitong pills.” It is also widely used as an ingredient in many products, *i.e.*, toothpaste, soap, and shampoo (Lu, Li & Wu, 2020; Lu et al., 2020). The germplasm resources of *Z. nitidum* in China are very rich. It can be divided into four types according to morphological characteristics, including three species (type I, II, and III) and one variety (type IV) (Qin et al., 2019a, 2019b).

Understanding the molecular markers of *Z. nitidum* will help identify all of its potential resources. The active components of *Z. nitidum*, including alkaloids, flavonoids, lignin, have already been isolated (Lu et al., 2020; Wang et al., 2022), but the biosynthetic pathways of the alkaloids have not been identified. Other genetic information about this species, including microsatellite marker characterization, is still lacking.

Next-generation sequencing (NGS) is a powerful tool for generating new and comprehensive sequence data of genetic resources, especially for species without reference genomes (Finotello & Di Camillo, 2015; Trombetta et al., 2014). However, because of short sequencing reads, fragmentation, and post-sequencing assembly, it is difficult to accurately obtain full-length transcripts and correct annotation information using NGS (Abdel-Ghany et al., 2016). Single molecule real-time (SMRT) sequencing can produce high-quality full-length transcripts, and analyze gene features such as gene families, long non-coding RNAs (lncRNAs), alternative splicing (AS) events, transcription factors (TF), and SSRs in medicinal plants and animals that lack reference genomes, such as *Cassia obtusifolia* (Deng et al., 2018), *Salvia miltiorrhiza* (Xu et al., 2015), *Fritillaria hupehensis* (Guo et al., 2021), *Artemisia argyi* (Cui et al., 2021), *Olea europaea* (Guodong et al., 2019), and *Gekko gecko* (Jiang et al., 2022). Candidate genes and biosynthesis pathways are identified using transcriptomes (Wang et al., 2020). SSR molecular markers are widely used to define alleles related to important agronomic traits, such as little millet (*Panicum sumatrense;* Desai et al., 2021), *Capsicum frutescens* (Zhong et al., 2021), garlic (*Allium sativum;* Li et al., 2022). However, to date, the full-length transcriptome sequence of *Z. nitidum* has not been generated.

In this study, SMRT sequencing was applied to generate the full-length transcriptome of *Z. nitidum*. Functional annotation was then performed based on the transcriptome data using publicly available databases and characterization of gene features, including lncRNA and TF prediction, and SSR analysis. In the absence of *Z. nitidum* reference genome, this can be used as a reference transcriptome for further genetic analyses. It will contribute to illustrate some important biological regulatory mechanisms, such as the regulation of alkaloid biosynthesis. Moreover, the SSRs identified in this study will promote the development of genetic markers for marker-assisted selection (MAS) in *Z. nitidum*.

## Materials and Methods

### Plant materials

All analytical methods were carried out in accordance with relevant guidelines and regulations. Fresh roots, leaves, flowers, fruits, old stems, and young branches were all collected from the same *Z. nitidum* (3-years old) plant in the Wuxu planting base (Nanning, Guangxi Province, China), immediately frozen in liquid nitrogen, and then stored at −80 °C until RNA extraction.

### RNA extraction

The total RNA of each sample was isolated using the Trizol RNA extraction kit (Invitrogen, Carlsbad, CA, USA) following the manufacturer’s instructions, then treated with RNase-free DNase I (TianGen, Beijing, China) to remove DNA contaminants.

The integrity and concentration of the RNA were evaluated using the Agilent Bioanalyzer 2100 system (Agilent Technologies, CA, USA) and a NanoDrop 2000 spectrophotometer (Thermo Fisher Scientific, Waltham, MA, USA). High-quality RNA of each sample was equally mixed as one pool for full-length transcriptome sequencing.

### Library construction and transcriptome sequencing

Full-length cDNA was synthesized from the purified total RNA using the SMARTer PCR cDNA Synthesis Kit (Takara Clontech Biotech, Dalian, China) following the manufacturer’s protocol, and large-scale PCR was conducted to produce more double-stranded cDNA templates. Size selection was then performed to generate SMRTbell™ libraries using a PacBio Template Prep Kit (PacBio, Menlo Park, CA, USA). Subsequently, full-length transcriptome sequencing of *Z. nitidum* was performed using the Pacific Sequel platform.

### SMRT sequencing data processing

Raw reads were processed into circular consensus sequencing (CCS) reads by adapting the PacBio SMRT analysis software v2.3.0 (https://www.pacb.com/products-and-services/analytical-software/smrt-analysis/). Full-length nonchimeric (FLNC) transcripts were determined and generated by searching for both the 5′ and 3′ cDNA primers and the poly A tail signal in CCS.

Consensus isoforms and full-length (FL) consensus sequences were determined using an iterative clustering for error correction (ICE) clustering analysis of FLNC. High-quality FL transcripts (identity >0.99) were acquired by removing redundant sequences using CD-HIT (Li & Godzik, 2006).

### Structure analysis and lncRNA prediction

Candidate coding regions of non-redundant transcript sequences were identified by TransDecoder (https://github.com/TransDecoder/TransDecoder/releases).

LncRNAs were identified based on the threshold of transcripts with lengths >200 nt using the predictor of long non-coding RNAs and messenger RNAs based on an improved k-mer scheme tool (PLEK), the coding potential calculator (CPC2), and the coding potential assessment tool (CPAT).

### Functional annotation

Non-redundant transcripts were functionally annotated using the following databases: nonredundant protein sequence database (Nr), Swiss-Prot database, TrEMBL database, Kyoto Encyclopedia of Genes and Genomes (KEGG), euKaryotic Ortholog Groups (KOG), Protein family (Pfam), and Gene Ontology (GO).

### Identification and characterization of SSRs and transcription factors

MIcroSAtellite (MISA) software (http://pgrc.ipk-gatersleben.de/misa/) was used to identify SSRs within the transcripts, and the characteristics of the repeated motif types were analyzed using the methods described by Feng et al. (2021). The TFs of *Z. nitidum* were identified using hmmsearch based on the Protein Family (Pfam) search results of the TF family.

### SSR validation

A set of 100 primer pairs were randomly selected and synthesized (Table S1). Leaf samples of thirty *Z. nitidum* trees from 10 different regions (*n* = 3), consisting of all of four types of trees, were collected from the Guangxi autonomous region and Guangdong Province, China. Detailed information about the samples collected are listed in Table S2. Genomic DNA was extracted using the cetyltrimethylammonium bromide (CTAB) extraction method as reported by Cheng et al. (2009). Polymerase chain reaction (PCR) was initiated with 1 μL template DNA (20 ng), 0.5 μL forward primers, 0.5 μL reverse primers, 5 μL 2× TaqPCR Master Mix and 3 μL sterile distilled water. PCR assay was conducted with the following condition: initial denaturation 95 °C for 5 min, denaturation 95 °C for 30 s, annealing 10 cycles 62–53 °C for 30 s, denaturation 95 °C for 30 s, annealing 30 cycles 52 °C for 30 s, and final extension at 72 °C for 20 min. Additionally, 21 tail sequences (5′-GAAGGTGACCAAGTTCATGCT-3′) of the forward primer of these SSR loci were added for detection by the ABI 3730XL DNA Sequencer. The polymorphism estimation, including polymorphism information content (PIC), observed heterozygosity (Ho), and expected heterozygosity (He) was then performed, followed by a cluster analysis based on the validated SSRs.

### Validation of transcript assembly

A total of 10 assembled transcripts were randomly selected to conduct validation using reverse transcription PCR (RT-PCR) amplification. The primers were designed based on the sequence of the *de novo* assembled transcripts (Table S3) by primer3. PCR amplification included initial denaturation at 95 °C for 5 min, 35 cycles of denaturation at 95 °C for 30 s, annealing at 60 °C for 30 s, extension at 72 °C for 30 s, final extension at 72 °C for 5 min, and storage at 4 °C. Finally, the amplified products were analyzed using agarose gel electrophoresis.

## Results

### PacBio single molecule long-read sequencing data analysis

The full-length transcriptome of *Z. nitidum* was obtained using PacBio SMRT sequencing technology. A total of 56.27 Gb of sequencing data were generated. After removing the adapter sequences, approximately 31,517,099 subreads remained with an average read length of 1,785 bp. To provide more accurate and reliable sequences, 456,109 CCSs with a mean length of 2,216 bp (Table 1), and 353,932 FLNC were generated.

**Table 1:** Summary of PacBio SMRT sequencing of *Z. nitidum*.

Category	Dataset
Subread base (G)	56.27
Subreads	31,517,099
Mean length of subreads	1,785
Reads of CCS	456,109
Mean length of CCS (bp)	2,216
FLNC reads	353,932
Mean length of FLNC (bp)	1,996
non-rerundant FLNC	16,555
Mean length of non-rerundant FLNC (bp)	2,058
Consensus transcripts	16,163
Mean length of consensus transcripts (bp)	1,171

**Figure 1:**
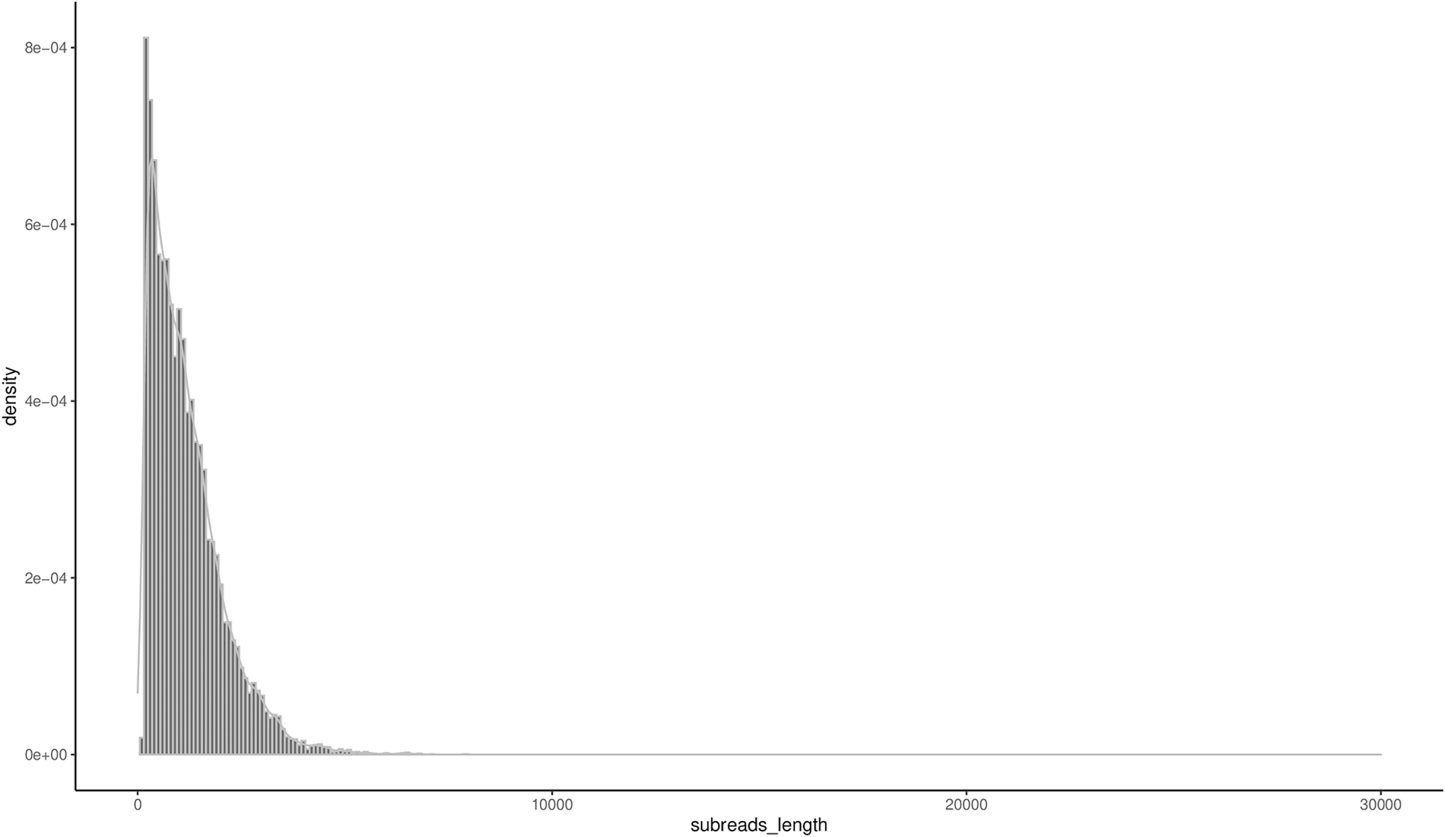
Distribution of the consensus transcripts.

A total of 16,555 high-quality FLNCs were obtained after clustering and removal of redundant sequences, and a subsequent analysis revealed 16,163 consensus transcripts for further annotation (Table S4). The length ranged from 147 bp to 7,917 bp, and the N50 and N90 were 1,617 bp and 621 bp, respectively. The mean transcript length was 1,171 bp (Table 2) with 51.49% of the transcripts less than 1,000 bp and 44.24% between 1,000–3,000 bp in length (Fig. 1).

**Table 2:** Statistics of FLNC in *Z. nitidum*. FLNC sequence refers to a class of full-length non chimeric CCS with 3′ end poly-A tails completely measured and no sequence chimerism.

Seq_number	Total_length	<1 kb	>1 kb & <2 kb	>2 kb & 3 kb	>3 kb	N50	N90	Mean	Median	Max	Min
16,163	18,928,494	8,323	5,391	1,759	690	1,617	621	1,171	972	7,917	147

### Functional annotation of transcripts

Of the 16,163 transcripts, 14,231 (88.05%) were successfully annotated against the Nr, Swiss-Prot, KEGG, KOG, GO, TrEMBL, and Pfam databases. The annotation rates were 14,199 (87.85%) in TrEMBL, 14,169 (87.66%) in Nr, 11,887 in Swiss-Prot (73.54%), 11,590 in Pfam (71.71%), 9,182 in KOG (56.81%), 7,684 in GO (47.54%), and 6,830 in KEGG (42.26%; Table 3).

**Table 3:** Statistics of annotation results. Adopt Siwss-Prot, Pfam, KEGG, GO, Nr, KOG, TrEMBL databases annotated the full-length transcripts.

Database	Annotated number	Annotated ratio/%
GO	7,684	47.54
KEGG	6,830	42.26
KOG	9,182	56.81
Nr	14,169	87.66
Pfam	11,590	71.71
Swiss-Prot	11,887	73.54
TrEMBL	14,199	87.85
Total	14,231	88.05

The GO analysis revealed that 7,684 unigenes were clustered into 33 GO terms, of which 52.22% unigenes were enriched in binding for molecular function, 47.39% unigenes were enriched in metabolic process, 27.51% unigenes were enriched in membrane parts.

**Figure 2:**
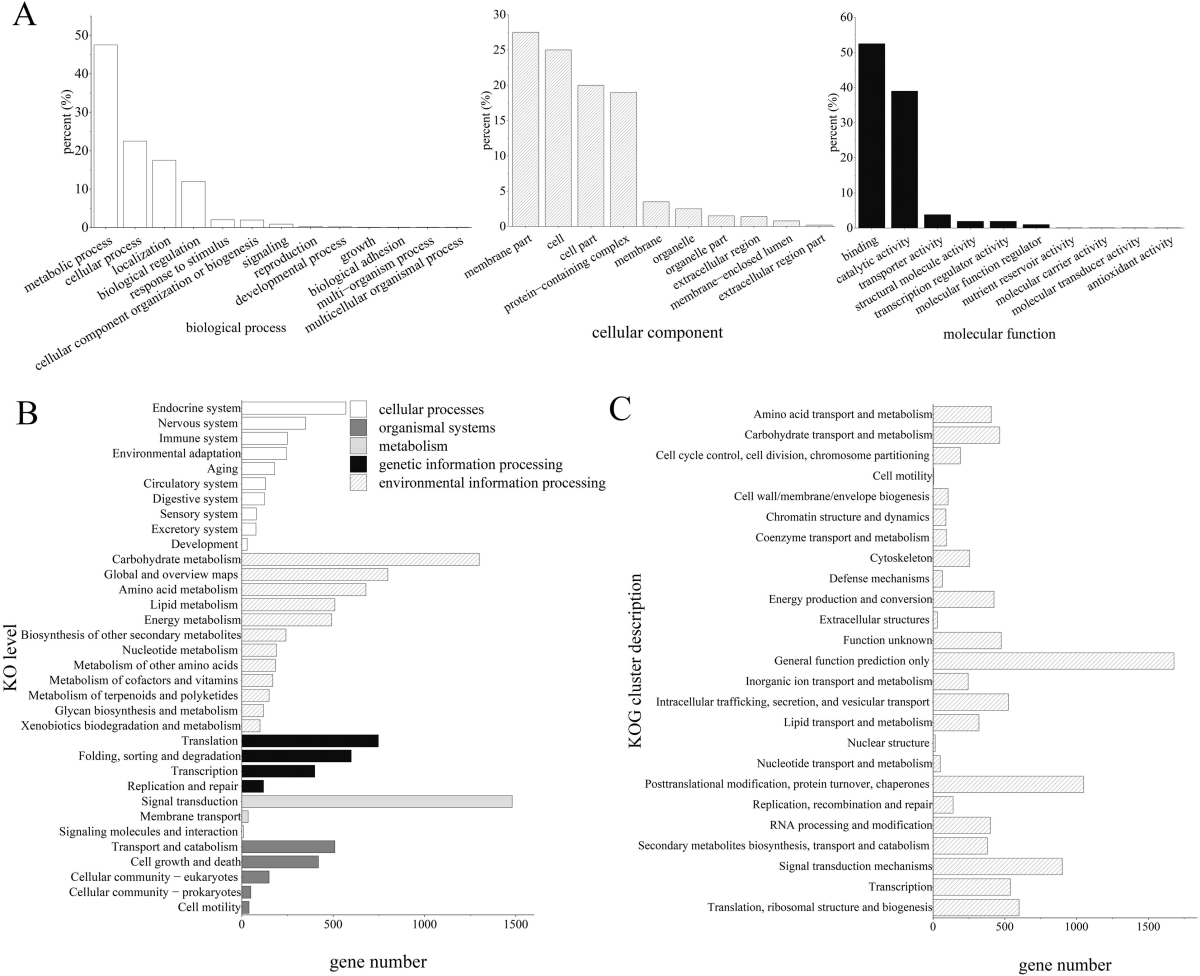
Functional annotation results of *Z. nitidum* transcripts. (A) GO annotation, (B) KEGG annotation, (C) KOG annotation.

Furthermore, the KEGG enrichment analysis classified 13,829 high-quality transcripts into 34 signaling pathways, which were involved in five categories: cellular processes (1,226, 7.58%), environmental information processing (1,525, 9.44%), genetic information processing (1,736, 10.74%), metabolism (5,048, 31.23%), and organismal systems (2,081, 12.88%; Fig. 2).

### Transcript validation

**Figure 3:**
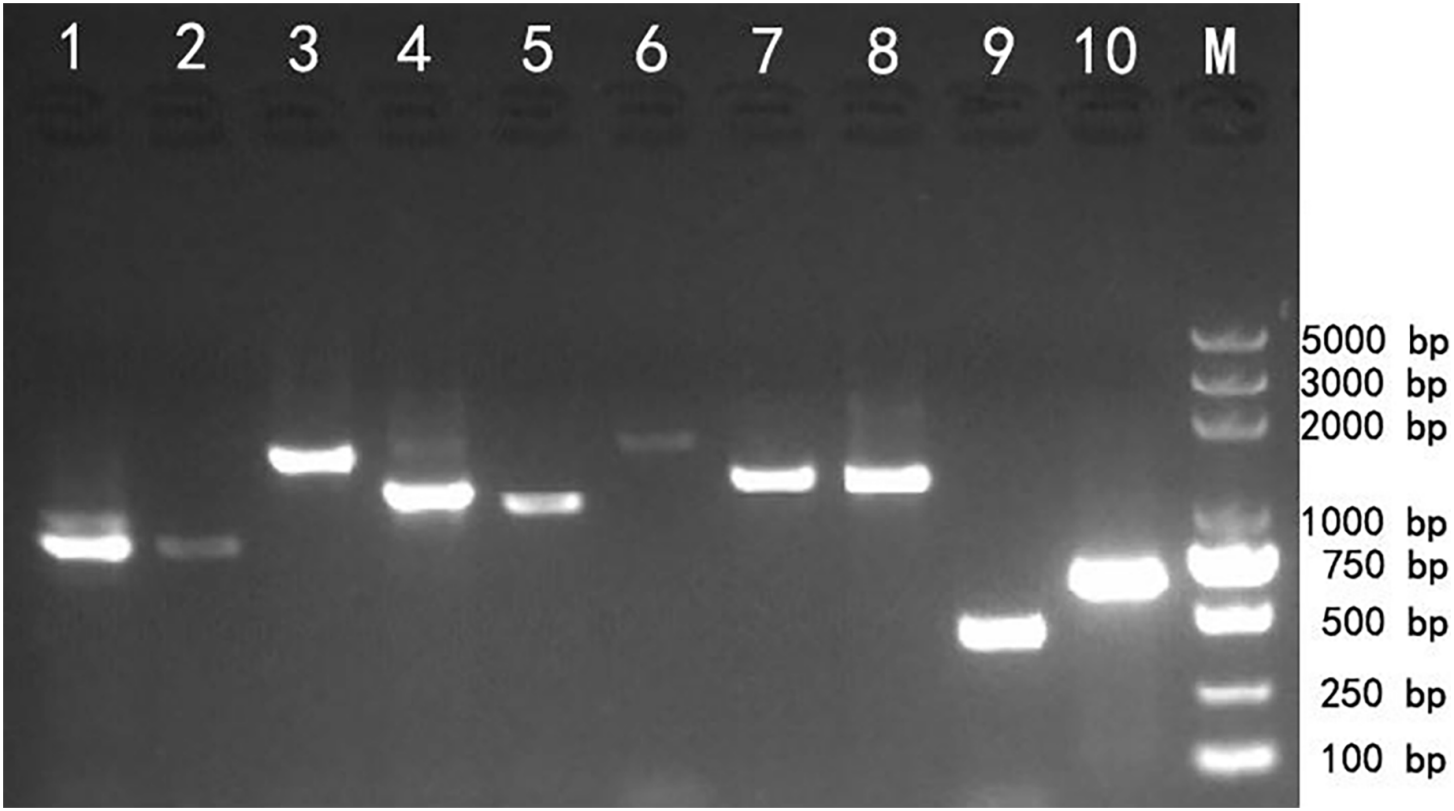
Reverse transcription PCR amplifcation of 10 selected genes from *Z. nitidum*.

Reverse transcription PCR (RT-PCR) amplification was used to validate the transcriptome assembly. The agarose gel analysis of the target products showed that the corresponding cDNA fragments were approximately the same as the expected size based on the transcript assembly, which demonstrated that the transcripts generated using SMRT technology could be reliably used for further gene identification and functional analysis (Fig. 3).

### Identification and characteristic analysis of lncRNAs

**Figure 4:**
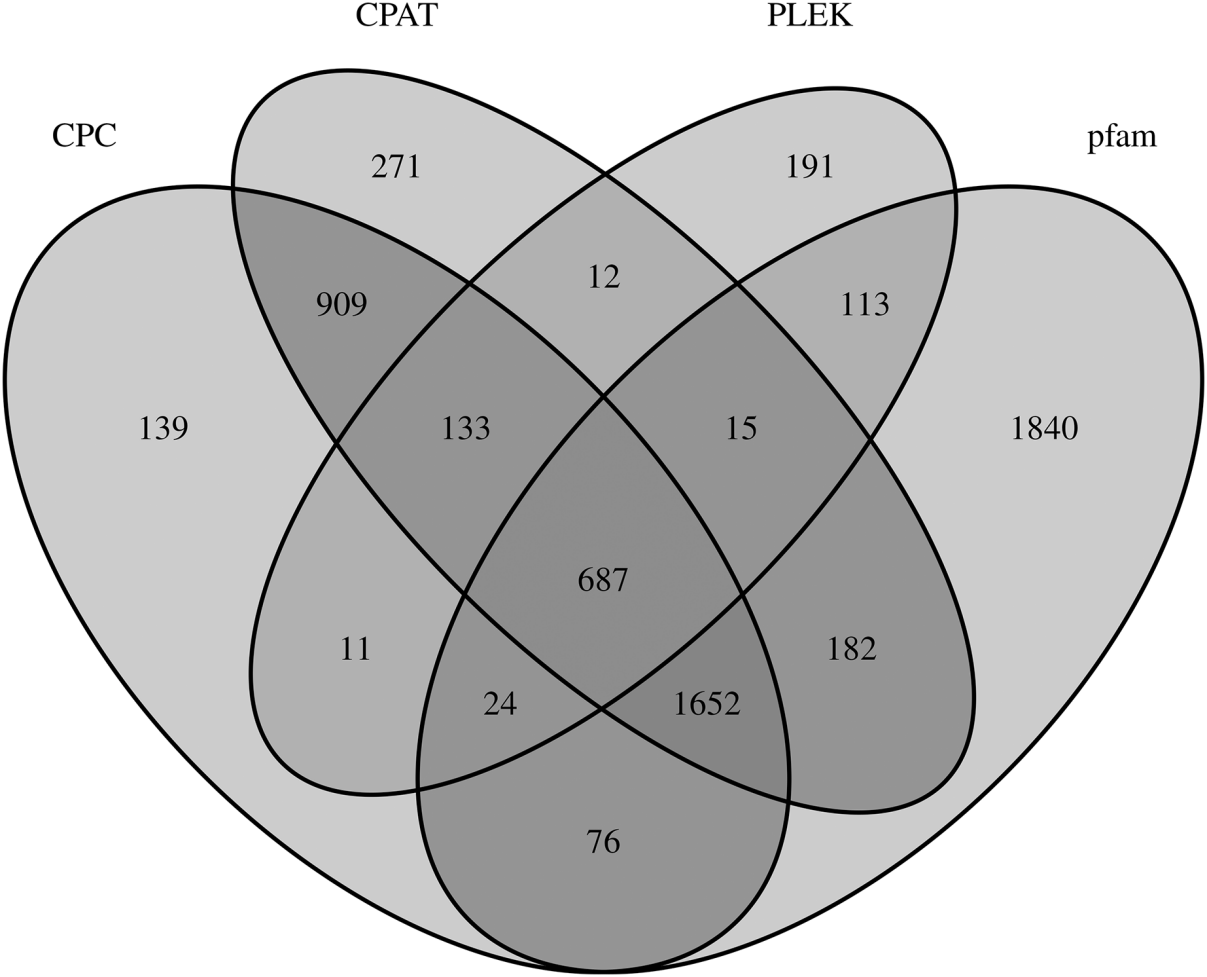
LncRNA prediction.

LncRNAs are RNA molecules those are more than 200 nucleotides in length which are not translated into protein. By filtering and excluding isoforms with lengths 200 nt, 3,631; 3,681; 1,186; and 4,589 lncRNAs were evaluated based on the CPC, CPAT, PLEK and Pfam databases, respectively. Only 687 of 6,255 total lncRNAs were found in all four computational approaches (Fig. 4).

### Transcription factor detection

**Figure 5:**
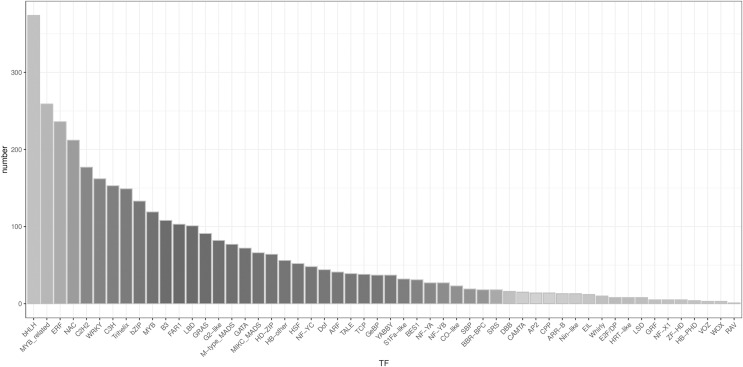
Type distribution of TFs.

A total of 3,482 TFs were identified. The most abundant TF families were the bHLH, MYB_related, ERF, and NAC families (Fig. 5).

### SSR detection

A total of 22,780 SSRs were identified using the MISA tool, including 13,800 mononucleotides (60.58%), 928 dinucleotides (4.07%). A total of 1,144 trinucleotides (5.02%), 53 tetranucleotides (0.23%), 14 pentanucleotides (0.06%), and 39 hexanucleotides (0.17%; Table 4).

**Table 4:** Statistical analysis of SSRs. Prediction of SSR sites in transcripts by MISA pl program.

Item	Number
Total number of sequences examined	16,163
Total number of sequences examined (bp)	517,279,084
Total number of identified SSRs	22,780
Number of SSR containing sequences	16,550
Number of sequences containing more than 1 SSR	4,687
Mononucleotides	13,800
Dinucleotides	928
Trinucleotides	1,144
Tetranucleotides	53
Pentanucleotides	14
Hexanucleotides	39

The number of repeat SSR motifs ranged from 5 to 496, with a mean of 147.432. We found that SSRs with 10 motif repeats were the most common and accounted for 7.04% (1,603) of all SSRs, followed by SSRs with 11 repeats (1,028, 4.51%), six repeats (764, 3.35%), and 12 repeats (728, 3.20%); 17,060 SSRs had motif repeat numbers ≥12, accounting for 74.89% of all SSR loci identified (Table S5).

**Figure 6:**
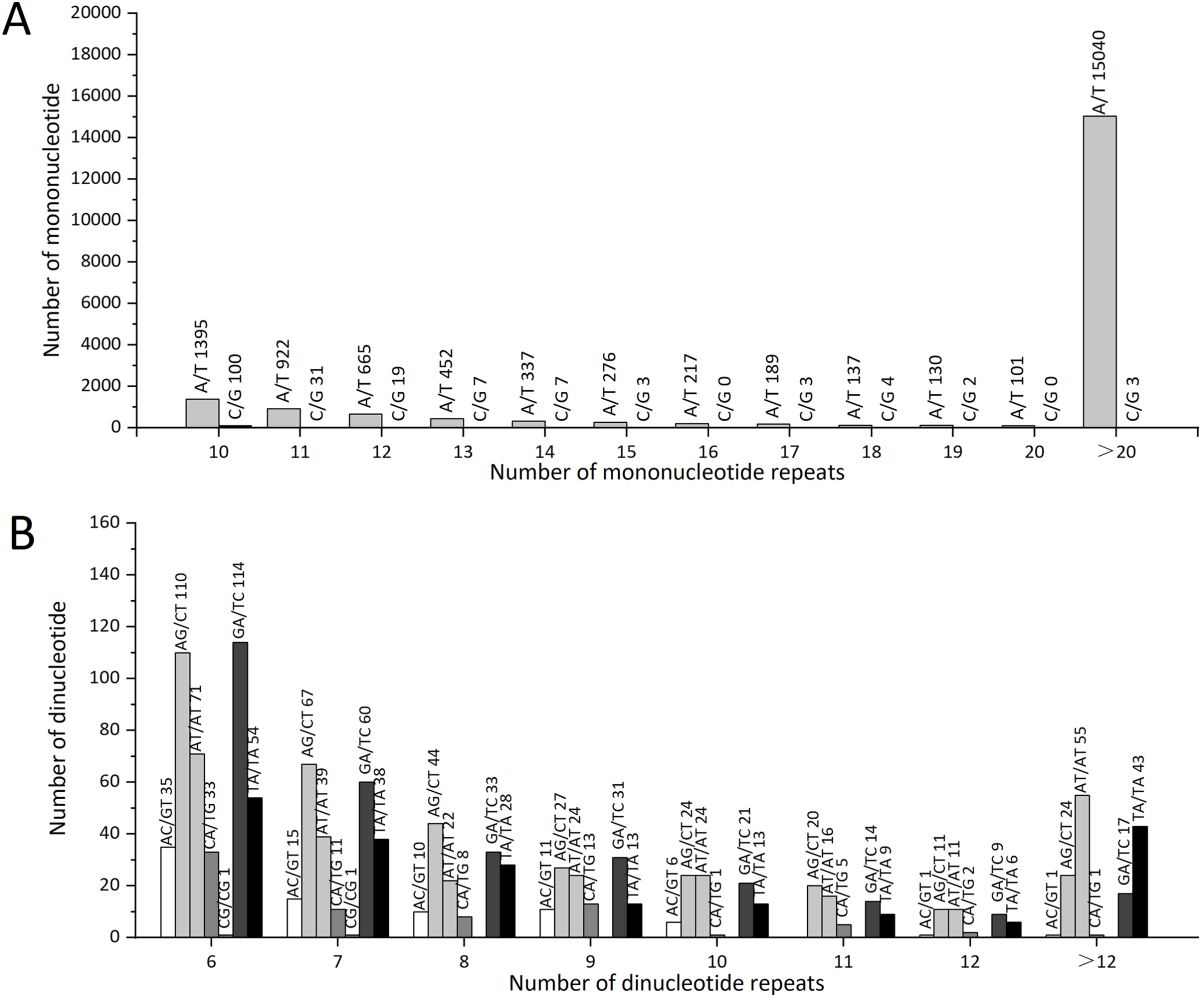
The types and numbers of mononucleotide and dinucleotide repeat motifs. (A) The number of mononucleotide repeats (B) The number of dinucleotide repeats.

The statistical analysis of all SSR loci showed that the repeat motif types with the highest numbers were: ATC/GAT (64), GAA/TTC (62), and AGC/GCT (51). In *Z. nitidum*, A/T was the most common mononucleotide repeat motif, accounting for 87.19% (19,861) of all mononucleotide repeats, while C/G represented only 0.79% (179; Fig. 6A). Of the dinucleotide repeats, the AG/CT motif was the most frequent (327, 1.44%), followed by GA/TC (299, 1.31%; Fig. 6B).

### SSRs validation

A total of 100 randomly-selected SSR primer pairs were chosen for verification. A total of 36 SSR primer pairs were successfully amplified and showed expected product size in all tested samples; 20 loci showed allelic polymorphism. The observed heterozygosity (Ho) ranged from 0.069 to 0.958, with an average of Ho = 0.478 (Table 5). A total of 126 alleles were obtained from 20 SSRs, and the number of alleles ranged from three to 16 per locus. Dominate alleles were found at the LMZ 51 locus, followed by LMZ73, LMZ96 1903, and LMZ 97. The average value of He was 0.674, but ranged between 0.494 and 0.916. The calculated PIC for each locus ranged from 0.382 to 0.916, with an average of 0.627.

**Table 5:** Novel genic SSR genetic diversity values in 30 *Z. nitidum* individuals.

SSR loci	Repeat motifs	Allele ranges (bp)	Ho	He	PIC	Na
LMZ33	(TC)10	248–261	0.571	0.737	0.704	8
LMZ34	(AG)6	297–364	0.958	0.646	0.599	6
LMZ37	(AG)8	270–279	0.103	0.6	0.523	5
LMZ44	(CTC)5	218–227	0.321	0.706	0.654	5
LMZ45	(TCT)6	195–211	0.621	0.693	0.642	5
LMZ46	(AAG)5	230–236	0.207	0.494	0.413	3
LMZ51	(CTT)11	272–295	0.759	0.916	0.91	16
LMZ58	(CCG)7	244–285	0.241	0.382	0.362	5
LMZ59	(CAG)6	202–214	0.345	0.578	0.534	5
LMZ60	(GCA)5	129–144	0.63	0.641	0.569	3
LMZ68	(GTG)5	185–195	0.321	0.581	0.519	4
LMZ71	(AAC)7	153–166	0.552	0.67	0.611	5
LMZ73	(ATA)19	208–257	0.429	0.765	0.745	10
LMZ76	(TAA)6	227–236	0.069	0.532	0.422	3
LMZ85	(TTTC)5	228–241	0.31	0.734	0.696	5
LMZ89	(AATC)7	165–179	0.483	0.644	0.6	6
LMZ94	(TCGGG)5	255–269	0.483	0.76	0.718	5
LMZ96	(CGCAGC)5	211–283	0.655	0.725	0.689	10
LMZ97	(CATGGG)5	229–282	0.75	0.83	0.811	9
LMZ98	(CCCAAG)5	122–164	0.75	0.845	0.825	8
Mean			0.478	0.674	0.627	6

**Figure 7:**
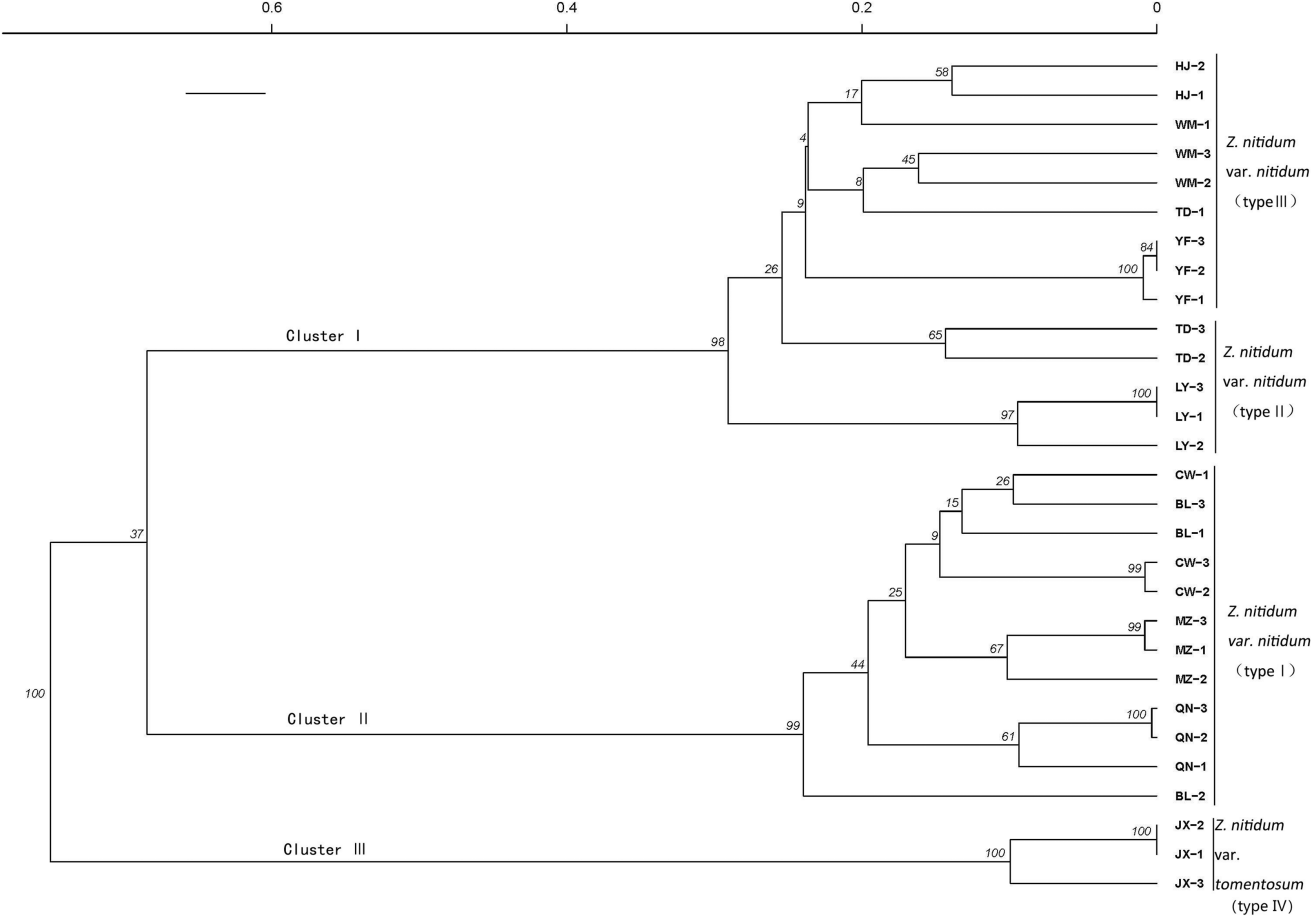
Genetic diversity analysis based on genic SSR markers.

A genetic correlation analysis was performed based on the verified SSR. The results showed three main clusters comprised of four total branches (Fig. 7). Cluster I had two branches, with branch one including nine genotypes belonging to type III and branch II including five genotypes collected from Baise, Guangxi province. Cluster II included 12 genotypes belonging to type I. Cluster III was comprised of three individuals belonging to type IV.

## Discussion

*Z. nitidum* has a long history of widespread use in traditional Chinese medicine and is used to treat conditions such as gastric ulcers, gastritis, and stomach cancer (Lu et al., 2020). The first transcriptome study of *Z. nitidum* only provided the candidate genes involved in the biosynthesis pathways for alkaloid, lignan, and avonoid (Wang et al., 2020). Genetic research of Liangmianzhen has been hampered by limited genetic resources. Acquiring a full-length transcriptome of *Z. nitidum* is the primary step to understanding gene function, so we used SMRT sequencing to perform full-length transcriptome sequencing of *Z. nitidum*.

We obtained a total of 56.27 Gb sequencing data. After quality control analysis, 456,109 CCSs, 353,932 FLNCs, and 16,163 consensus transcripts with an average read length of 1,171 bp were aquired. The length of N50 was 1,617 bp, which was longer than that was found by Wang et al. (2020). High-quality transcripts were annotated using seven public databases, and the annotated transcript accounted for 88% of all transcripts, a similar rate to transcriptomics studies of *Pinctada fucata martensii* (Zhang et al., 2020) and Mangrove Clam *Geloina erosa* (Liao et al., 2022), but higher than reported by Wang et al. (2020). This indicates that the higher read lengths of SMRT sequencing compared to transcriptome sequencing increases annotated transcript percentages (Zhang et al., 2020).

GO classification revealed that the majority of the unigenes were involved in the multicellular organismal process, the multi-organism process, reproduction, and biological adhesion. KOG classification indicated that the major transcripts were associated with general function prediction, posttranslational modification, protein turnover, chaperones, and signal transduction mechanisms. The KEGG analysis demonstrated that 1,488, 1,293, and 781 transcripts were assigned to signal transduction, carbohydrate metabolism, and global and overview maps, respectively. We used RT-PCR to verify the assembled transcript, and the results demonstrated that the transcriptome assembly in our study was reliable and could be used for further research.

LncRNAs function as transcriptional regulators by binding to TFs to modulate gene expression (Long et al., 2017). Research on the regulatory mechanism between lncRNA and TF in plants has increased considerably in the last decade (Moison et al., 2021; Zheng et al., 2022), but no lncRNAs and TFs have been identified among the *Z. nitidum* transcripts. In our study, 6,255 lncRNAs and 3,482 TFs were predicted, with bHLH identified as the dominant transcription factor. Basic helix-loop-helix (bHLH) families have a wide array of functions, which are involved in diverse regulatory networks by interacting with target genes to modulate biosynthesis, metabolism, and transduction of plant hormones (Feller et al., 2011). Our results will accelerate further research on the regulatory network of these lncRNAs and TFs in the *Z. nitidum* transcriptome.

Molecular markers, such as SSR, SNP, and indel, are widely used in molecular identification, molecular marker-assisted selection, and genetic selection in animals and plants (Jiang et al., 2019; Zhang et al., 2022). In this study, we detected 22,780 SSRs in 16,163 sequences. Among them, mononucleotides accounted for 61.80%. The most frequent type of mononucleotide, dinucleotide, or trinucleotide repeat motifs were A/T, AG/CT, and GAA/TTC. These results are the same as the most frequent repeat motifs in *Paulownia catalpifolia* (Feng et al., 2021), Chinese cabbage (Song et al., 2015), and *Rhododendron lapponicum* (Jia et al., 2020), while studies on *Prunus virginiana* and *Rhododendron lapponicum* suggest that CT participates in regulation transcription and expression (Jia et al., 2020; Wang et al., 2012).

In order to validate the SSRs, 36 out of 100 primer pairs were positively amplified, with 20 (55.6%) showing polymorphism in 30 individuals among 10 batches of *Z. nitidum*, which is higher than the results reported by Duan et al. (2017). The failed amplification of the SSRs might be related to the lack of a reference genome, and highly repetitive sequences (Varshney, Graner & Sorrells, 2005). Additionally, due to the greater depth of sequencing coverage, the average PIC was greater than the average PIC of 0.6573 recorded by Desai et al. (2021). A high level of genetic variability (average HO = 0.478, HE = 0.674) was also observed.

A cluster analysis grouped genotypes into three clusters consisting of four branches. The SSRs identified in our study could be used for molecular marker development to further molecular breeding in *Z. nitidum*.

## Conclusions

A high-quality, full-length transcriptome of *Z. nitidum* was acquired using the PacBio SMRT sequencing platform and 16,163 transcripts, 6,255 lncRNAs, 3,482 TFs, and 22,780 SSRs were identified. These results provide a comprehensive genome for further gene annotation and gene structure analysis. Furthermore, 20 SSRs showed polymorphism across the populations of *Z. nitidum*. Our findings provide a valuable resource for further genetic research on the molecular markers, molecular events, and regulatory networks of *Z. nitidum*.

## Supplemental Information

10.7717/peerj.15321/supp-1 Supplemental Information 1 100 pairs of SSR primers.

10.7717/peerj.15321/supp-2 Supplemental Information 2 Detail information of of tested *Z. nitidum* resources.

10.7717/peerj.15321/supp-3 Supplemental Information 3 10 pairs of primers with significant polymorphism.

10.7717/peerj.15321/supp-4 Supplemental Information 4 Transcript Details.

10.7717/peerj.15321/supp-5 Supplemental Information 5 The six types of SSR repeat motifs and their frequency in *Z. nitidum*.

## Additional Information and Declarations

### Data Availability

The following information was supplied regarding data availability: The raw sequence data are available at the Genome Sequence Archive in the National Genomics Data Center, China National Center for Bioinformation/Beijing Institute of Genomics, Chinese Academy of Sciences: GSA: CRA008080.
