# Supplementary material for: Full-length transcriptome analysis of Zanthoxylum nitidum (Roxb.) DC
Source: PeerJ. 2023 May 4;11:e15321. doi: 10.7717/peerj.15321 (PMC10164372; doi:10.7717/peerj.15321)
Supplement: Supplemental Information 2 [file peerj-11-15321-s002.doc]

| **Sample name** | **Collection location** | **species** |
| --- | --- | --- |
| BL | Beiliu, Yulin, Guangxi | *Zanthoxylum nitidum* var. *nitidum* （type 1） |
| CW | Cangwu, Wuzhou, Guangxi | *Zanthoxylum nitidum* var. *nitidum* （type 1） |
| HJ | Huanjiang,Hechi, Guangxi | *Zanthoxylum nitidum* var. *nitidum* （type 3） |
| JX | Jin Xiu, Laibin, Guangxi | *Zanthoxylum nitidum* var. *tomentosum* |
| LY | Lingyun, Baise,Guangxi | *Zanthoxylum nitidum* var. *nitidum* （type 2） |
| MZ | Pingyuan, Meizhou, Guangdong | *Zanthoxylum nitidum* var. *nitidum* （type 1） |
| QN | Qinnan, Qinzhou, Guangxi | *Zanthoxylum nitidum* var. *nitidum* （type 1） |
| TD | Tiandeng, Baise,Guangxi | *Zanthoxylum nitidum* var. *nitidum* （type 2） |
| WM | Wuming, Nanning, Guangxi | *Zanthoxylum nitidum* var. *nitidum* （type 3） |
| YF | Yuncheng, Yunfu, Guangdong | *Zanthoxylum nitidum* var. *nitidum* （type 3） |

**Table S2 Detail information of of tested Z. nitidum resources**
