# Supplementary material for: Full-length transcriptome analysis of Zanthoxylum nitidum (Roxb.) DC
Source: PeerJ. 2023 May 4;11:e15321. doi: 10.7717/peerj.15321 (PMC10164372; doi:10.7717/peerj.15321)
Supplement: Supplemental Information 3 [file peerj-11-15321-s003.doc]

**Table S3 10 pairs of primers with significant polymorphism**

| **Gene name** | **CDS area length** | **Primer sequence (5'-3')** | **Product size** |
| --- | --- | --- | --- |
| >transcript/15697 | 1536 | F:GAGCCTCAATTCTGATAGAAG R:AGATGGTCGCTGGCCTCTCACAAGA | 1596 |
| >transcript/16489 | 1212 | F:CATCAGTCTTGCGTGCATATATCC R:GTAAAAAGCCTAGCAATAATAATCC | 1470 |
| >transcript/21932 | 1176 | F:GGATTAGTTTGTCAGCAGAAAT R:TATAGCTAGTACTGTGTCGTAGACT | 1221 |
| >transcript/24621 | 1173 | F:CGTCGATGAATACCACAAGGCTC R:CTAAAGTGGAAAGGGAATCAAATGG | 1200 |
| >transcript/27386 | 1068 | F:CACCGCCACATGCACAGGCCAAG R:CATGGACGTGCTTCTCTCTTCAATA | 1144 |
| >transcript/28816 | 990 | F:GAGGGCATATCACTTGACATTATT R:CACCTTTTATTATTTTTGCTTAAAT | 1035 |
| >transcript/30172 | 765 | F:GGGGGTTGATCTTGATGCTGTGG R:TCAATGAGCCATTGTTGATCGC | 825 |
| >transcript/34043 | 771 | F:GGGAGGATCTTTTCATTCGAG R:TACCTACTAAAAATCGACTACCAAT | 808 |
| >transcript/34453 | 570 | F:GAGAGGATTGGTGATTGAAGGC R:GATTAAAACGCTGCCGTCTCTTATT | 616 |
| >transcript/41422 | 372 | F:GAGGGTATACGATACCCAAAGG R:AGCTAGCAACCATTCCTGAACCAAG | 419 |
